# Quality of life after definitive linear accelerator-based stereotactic radiotherapy for prostate cancer: a longitudinal study

**DOI:** 10.1186/s13014-022-02061-y

**Published:** 2022-05-12

**Authors:** Hideomi Yamashita, Mami Ogita, Subaru Sawayanagi, Yuki Nozawa, Osamu Abe

**Affiliations:** Department of Radiology, The University of Tokyo Hospital, 7-3-1 Hongo, Bunkyo-ku, Tokyo, 113-8655 Japan

**Keywords:** Stereotactic body radiotherapy, Prostate cancer, Quality of life, Patient-reported outcomes, Radiation therapy

## Abstract

**Background:**

Prostate cancer is the second most common malignancy worldwide, and the majority of patients are diagnosed with localized disease. We examined patients’ quality of life after stereotactic body radiation therapy (SBRT) for prostate cancer.

**Methods:**

We included patients who were treated between 2016 and 2020. Inclusion criteria were adenocarcinoma of the prostate; class risk of low, intermediate, and high; and a World Health Organization performance status of 0–2. Quality of life was measured using the Functional Assessment of Cancer Therapy-Prostate (FACT-P).

**Results:**

A total of 439 patients were treated with SBRT, with a median age of 73 years old. The median follow-up period was 34 months. FACT-P Trial Outcome Index (*p* < 0.0001), FACT-General (*p* = 0.0003), and FACT-P-Total (*p* < 0.0001) scores declined at 1 month post-SBRT, then recovered and returned to the same level as before treatment at 3–4 months post-SBRT. The decrease in quality of life in the first month was particularly remarkable in patients who received long-term hormone injections (36%). One month after the end of SBRT, about 22% of patients experienced "quite a bit” or more troubling side effects.

**Conclusions:**

This study showed longitudinal changes in quality of life by FACT-P after SBRT for prostate cancer. Overall, prostate SBRT was well tolerated.

## Introduction

Prostate cancer (PC) is the second most common malignancy worldwide, and the majority of PC patients are diagnosed with localized disease [1]. There are multiple efficacious guideline-recommended treatment options for localized PC, including radical prostatectomy, external beam radiation, and brachytherapy. Recent American Society for Radiation Oncology/American Society of Clinical Oncology/American Urological Association guidelines [2] included recommendations regarding the use of stereotactic body radiotherapy (SBRT) for PC. According to the National Comprehensive Cancer Network (NCCN) Guidelines Version 3.2022 [3], SBRT combined with androgen deprivation therapy can be considered if delivering longer course of external beam radiotherapy would present medical or social hardship for unfavorable intermediate, high, and very high risk. There is growing consciousness that quality of life (QoL) with objective measurement of late adverse genitourinary (GU) and gastrointestinal (GI) toxicity are indispensable for the treatment decision of patients with PC [4–6]. In Japan, SBRT for localized PC was covered by insurance in 2016, which is relatively recent. Health-related QoL is an increasingly important endpoint in PC care. Although the QOL fluctuations in the SRT for PC of Japanese are a very important theme, there are no reports on it yet. To the best of our knowledge, there have been no reports evaluating QoL fluctuations after radical prostate SBRT using the Functional Assessment of Cancer Therapy-Prostate (FACT-P) questionnaire. Therefore, the present study investigated the long-term QoL of patients after SBRT using a valid and self-administered QoL questionnaire via the FACT-P.

## Methods

We conducted a retrospective study of patients with PC treated with SBRT at our institution (University of Tokyo Hospital). For clinical evaluation, all patients who underwent RT for prostate cancer were asked to fill out a FACT-P QOL questionnaire immediately before SRT and at each outpatient follow-up after SRT. This time, retrospectively, only consecutive cases that satisfy the conditions described below were selected from them, and the results of the data were summarized in this study. Staging examinations included serum prostate-specific antigen (PSA) measurement, ultrasound, digital rectal exam, pelvic magnetic resonance imaging (MRI), prostate biopsy, chest/abdominal/pelvic computed tomography (CT), and technetium-99 m-methylene diphosphonate bone scan. The inclusion criteria were as follows: (1) histologically proven adenocarcinoma of the prostate; (2) NCCN class risk by NCCN Guidelines Version 1.2021 of low, intermediate, or high [3]; and (3) World Health Organization (WHO) performance status of 0–2 [7]. The exclusion criteria were as follows: (1) lymph node metastasis (n = 12), (2) bone metastasis (n = 11), (3) castration-resistant PC (n = 7), (4) after radical prostatectomy (n = 1), (5) SBRT for local recurrence after external beam radiation therapy (n = 2), (6) local recurrence after high-intensity focused ultrasound (HIFU) (n = 3), and (7) receiving more or less than five fractions of radiotherapy (n = 1). We did not limit the prostate volume or baseline International Prostate Symptom Score to maximize the number of patients included. The nodal involvement risk was not assessed in this paper, and it was not considered as exclusion criteria.

### Combination hormone therapy

Hormone therapy was administered over a short term of 4–6 months in the intermediate -risk group, and over a longer term of 1.5–2 years in the high-risk group. There was no concomitant use of bicalutamide, the first Degarelix injection, and the second and subsequent leuprorelin injections. SBRT was performed when PSA dropped to near 1.0 mg/mL. However, SBRT was started by the second month in the short-term administration group and by half a year in the long-term administration group.

### SBRT method

The clinical target volume (CTV) included the prostate and the proximal 2 cm of the seminal vesicle (SV) for high-risk patients, the prostate and 1 cm of the SV for intermediate-risk patients and the prostate only for low-risk patients. European Organization for Research and Treatment of Cancer (EORTC) guideline was used as a reference for our CTV delineations [8]. MRI imaging was used for delineation of the CTV. The planning target volume (PTV) consisted of a CTV with a posterior 3 mm margin and a 5 mm margin for all other margins [9].

The irradiation dose was PTV 95% prescription. 36.25 Gy in five fractions was 54%, and 40 Gy in five fractions was 43%. The former had been adopted before July 2018 and the latter after that. SBRT was performed using volumetric modulated arc therapy (VMAT) with flattening filter-free beams using a linear accelerator with image-guidance. Image guidance was performed using daily cone-beam computed tomography (CBCT). SBRT was performed five times every other day, excluding weekends. There were many cases of 177 patients in which SpaceOAR hydrogel (SpaceOAR; Augmenix Inc.) was applied before SBRT. Our previous report [10] describes the spacer insertion method, MRI planning, CT scan for SBRT, definition of the target volume, and the organs at risk. Each patient was given a glycerin enema of 30 or 40 cc 2 h before the examination or SBRT. After that, the patients were asked to drink water and hold their urine for 2 h. From 4 days before the examination to the last day of SBRT, the patients were encouraged to take elobixibat hydrate or polyethylene glycol daily. SBRT dose constraints are shown in Table 1. The Georgetown University experience was used as a reference for our dose constraints [11].

**Table 1:** Dose constraint of 40 Gy in 5 fractions

Dose constraint		With SpaceOAR		Without SpaceOAR	
Prostate	Volume	Goal	Tolerance	Goal	Tolerance
Urethra	Max	< 42.6 Gy	< 43.0 Gy	< 42.6 Gy	< 43.0 Gy
PTV	V110%	< 0.5%	< 2%	< 0.5%	< 2%
	D99%	> 39.5 Gy	> 39.17 Gy	> 39.2 Gy	> 38.1 Gy
	Max	< 43.26 Gy	< 44.14 Gy	< 43.9 Gy	< 45.5 Gy
	Mean	< 41.16 Gy	< 41.71 Gy	< 41.5 Gy	< 42 Gy
Bladder	V40Gy	< 3.7%	< 6%	< 4.6%	< 6%
	V20Gy	< 22.8%	< 35%	< 27.4%	< 49%
	V40.83 Gy	< 6.2 cc	< 8 cc	< 6.9 cc	< 10 cc
	Max	< 42.7 Gy	< 44.14 Gy	< 43.4 Gy	< 45 Gy
	Mean	< 11.92 Gy	< 16.55 Gy	< 13.7 Gy	< 20 Gy
Rectum	1 cm above and below the PTV				
	V40Gy	< 0.5%	< 1.5%	< 2.5%	< 4%
	V36Gy	< 2.5%	< 5.7%	< 8%	< 11%
	V32Gy	< 5%	< 10%	< 13%	< 17%
	V30Gy	< 6.8%	< 13%	< 16%	< 20%
	V20Gy	< 28.2%	< 34%	< 32%	< 39%
	Max	< 40.83 Gy	< 42.48 Gy	< 42.7 Gy	< 43.9 Gy
	Mean	< 16.88 Gy	< 18.21 Gy	< 17.4 Gy	< 19.6 Gy
Femoral head	Max	< 17.66 Gy	< 18.76 Gy	17.66 Gy	< 18.76 Gy
Sigmoid colon	V30Gy	< 0 cc		< 1 cc	
Penile bulb	Max	< 40 Gy		< 40 Gy	
Small bowel	V30Gy	0 cc	< 5 cc	0 cc	< 5 cc

### Description of the patient cohort

Baseline patient characteristics are shown in Table 2. The median follow-up period was 34 months (maximum: 54 months). Between May 2016 and December 2020, 439 patients answered the FACT-QoL questionnaire. These 439 patients were all that had been treated with SBRT for PC with curative intent at our institution during this period. The median age of the sample was 73 years and the median PSA before all treatments was 9.2 ng/mL. Clinical stage T2a was the most prevalent T-stage, with 47% of cases diagnosed as such. Regarding Gleason Score, 28% and 27% of patients were classified as Group 2 and 3, respectively. Moreover, 53% of patients were classified as intermediate-risk, while 42% were classified as high-risk. Short-term hormones were given for 4–6 months to 42% of patients, while long-term hormones were given to 36% of patients.

**Table 2:** Patient characteristics

Factors	N	%
Total	439	100
Age (years old)		
Median (range)	73 (33–92)	
Quartile	68 and 78	
iPSA (ng/mL)		
Median (range)	9.2 (1.6–24.0)	
Quartile	6.3 and 14.7	
Clinical T-stage		
T1c	84	19.1
T2a	208	47.4
T2b	20	4.6
T2c	61	13.9
T3a	46	10.5
T3b	14	3.2
T4	6	1.4
Gleason score group		
Group 1	46	10.5
Group 2	121	27.6
Group 3	117	26.7
Group 4	82	18.7
Group 5	73	16.6
Risk group		
Low	21	4.8
Intermediate-low	84	19.1
Intermediate-high	149	33.9
High	112	25.5
Ver-high	73	16.6
Hormonal therapy		
None	98	22.3
Short term	183	41.7
Long term	158	36.0
RT total dose		
36.25 Gy	239	54.4
40 Gy	187	42.6
42.5 Gy	13	3.0

*PSA* prostate-specific antigen, *RT* radiation therapy

A total of 177 patients (40%) had a hydrogel spacer inserted between their prostate and rectum before SBRT. Regarding baseline characteristics of patients with SpaceOAR application, 36.25 Gy, 40 Gy, and 42.5 Gy were 55%, 39%, and 6%, and low, intermediate, and high-risk were 7%, 52%, and 40%, and short-term and long-term combined hormones were 40% and 31%, respectively.

### Follow-up

Follow-up intervals were calculated from the date of the last SBRT dose. Outpatient follow-up was conducted at 1 month and 3–4 months after the end of SBRT, then at every 3 months until 2-year follow-up, and finally at every 6 months after the second year. We asked patients to complete a QoL questionnaire at each follow-up visit from May 2016. PSA was measured at every visit, and when it exceeded 2.0 ng/mL twice in a row, contrast-enhanced MRI and/or 18F-fluorodeoxyglucose (FDG) positron emission tomography (PET) examination was added to check for recurrence, since prostate specific membrane antigen (PSMA) was not be approved at the moment in Japan. When treatment such as chemotherapy was started in the case of double cancers, it was excluded from the survey.

### Statistical analysis

Risk classification was based on the NCCN risk classification v1. 2021 for PC. The paired t-test was used for comparison of the baseline. All *p*-values were two-sided, and *p* < 0.05 was considered statistically significant. The FACT-P instrument is a multidimensional, self-administered, 39-item questionnaire. A FACT-P total score (FACT-P-Total) was obtained by adding together each patient’s emotional well-being (EWB), family well-being (FWB), physical well-being (PWB), social well-being (SWB), and prostate cancer subscale (PCS) (range 0–152) scores. The FACT-General (FACT-G) total score by determined by adding together the EWB, FWB, PWB, and SWB (range 0–104) scores. The FACT-P Trial Outcome Index (TOI) was determined by adding the FWB, PWB, and PCS subscale scores (range 0–104). Scores were obtained according to version 4 of the FACT scoring guidelines [12]. Prorating was carried out only if more than 50% of the items on each subscale were answered. For all scales, the higher the score, the better the QoL. This study was approved by the Institutional Review Board of Tokyo University (No. 3372–6). All methods were performed in accordance with the relevant guidelines and regulations. Informed consent was obtained from all participants.

## Results

The baseline prostate volume was 39.3 cc (SD: 20.9) and the baseline international prostate symptom score was 9.9 (SD: 1.5).

**Fig. 1:**
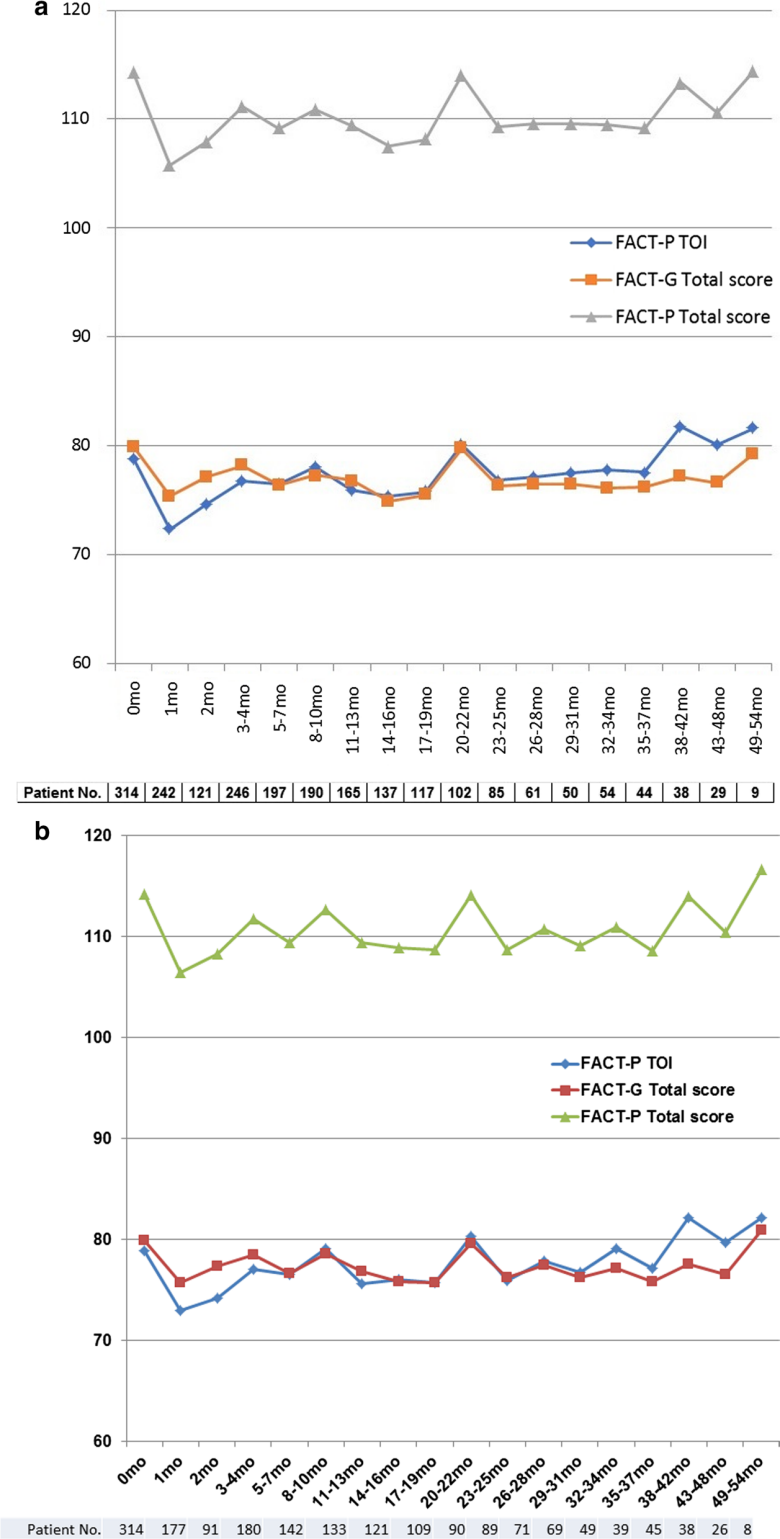
Mean Functional Assessment of Cancer Therapy-Prostate (FACT-P) Trial Outcome Index score (TOI, blue line), Functional Assessment of Cancer Therapy-General (FACT-G) Total score (orange line), and FACT-P-Total score (gray line). Time from last SBRT dose (months). For all patients (**a**) and excluding 125 cases who did not respond to the questionnaire before SBRT (**b**)

Since we included all patients received SBRT in this survey, 28.5% of patients did not complete the questionnaire before treatment. In other words, although the questionnaire was distributed to all 439 patients, 314 patients (71.5%) answered it before treatment and 242 (55%) at one month. Even after that, the questionnaire continued to be distributed to all 439 patients every time they came to the outpatient department, everyone answered it at least once at some point. The number of the returned questionnaires at each point was shown in the bottom of Fig. 1. Across the FACT P-TOI, FACT-G, and FACT-P-Total, the QoL score at 1 month after the end of SBRT was significantly lower than that before SBRT (Fig. 1a). The average QoL score dropped from 78.84 (standard error: 0.77) before SBRT to 72.35 (1.02) at 1 month in the FACT P-TOI (*p* < 0.0001 by t-test), from 79.91 (0.79) to 75.38 (0.96) in the FACT-G (*p* = 0.0003), and from 114.22 (1.05) to 105.78 (1.31) in the FACT-P-Total (*p* < 0.0001). An improving trend was then seen in the second month (average score: 74.62 [SE: 2.57], 77.13 [2.09], and 107.88 [3.23], respectively). Furthermore, at 3–4 months, the scores of the FACT P-TOI (*p* = 0.0011, average score: 76.80 [SE: 0.88]), FACT-G (*p* = 0.0410, 78.15 [0.94]), and FACT-P-Total (*p* = 0.0029, 111.19 [1.24]) improved significantly when compared with those recorded in the first month. There was no significant decrease in the QoL score during the subsequent follow-up period, until months 49–54 (Fig. 1a). Figure 1b shows the QOL fluctuations excluding 125 cases who did not respond to the questionnaire before SBRT. The average QoL score dropped to 72.92 (SE: 1.15) at 1 month in the FACT P-TOI, 75.77 (1.11) in the FACT-G, and 106.46 (1.49) in the FACT-P-Total. The fluctuations are almost the same as those in Fig. 1a.

**Fig. 2:**
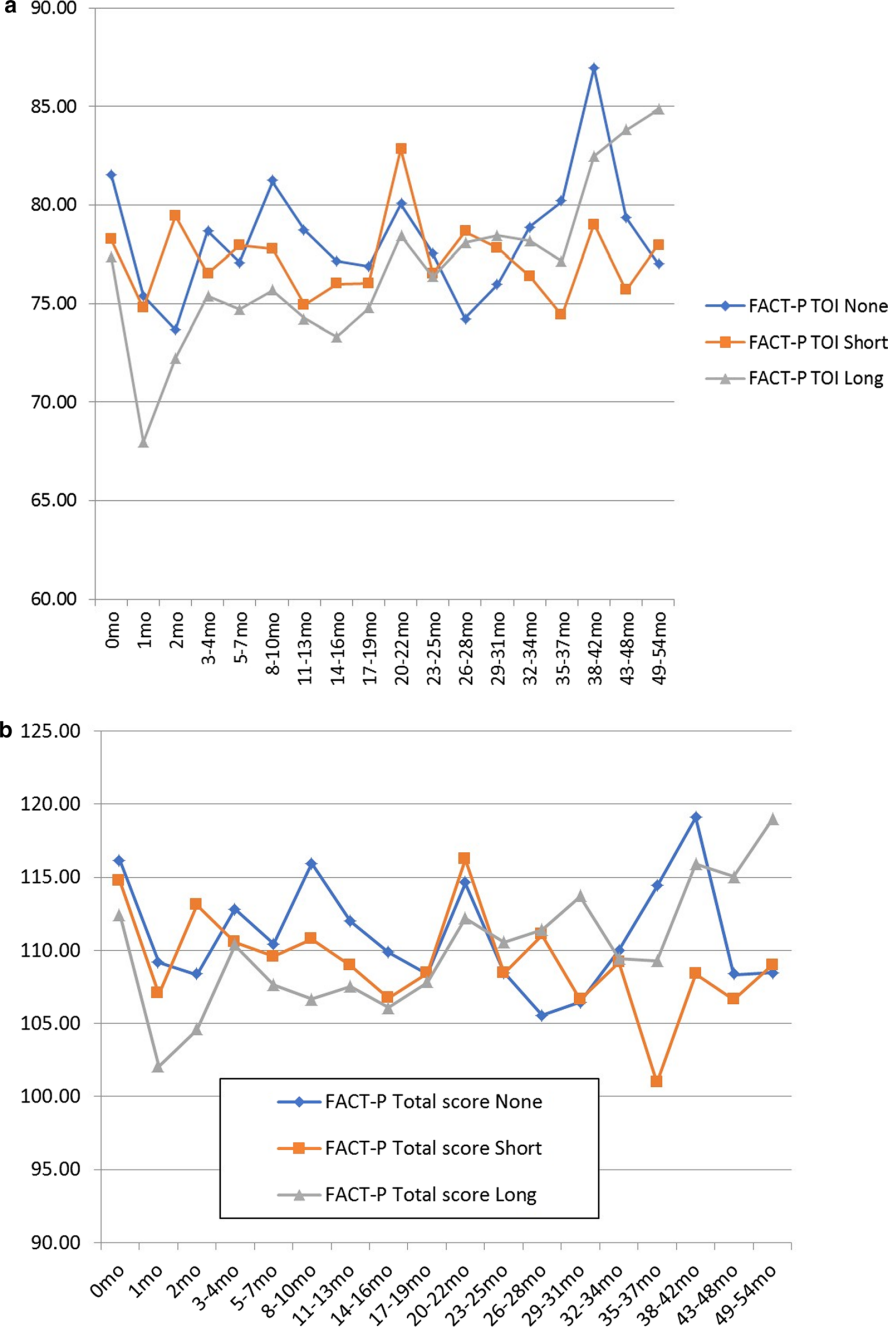
Mean Functional Assessment of Cancer Therapy-Prostate (FACT-P) Trial Outcome Index (TOI) score (**a**) and mean FACT-P-Total score (**b**) by no (blue line), short term (orange line), and long term (gray line) hormonal therapy

In the long-term hormone group, the QoL reduction which was seen in the FACT P-TOI (67.99) and FACT P-Total (102.08) at 1 month was larger than those in the short-term hormone group and the hormone-free group (Fig. 2). In the hormone-free group, QoL was lowest at two months instead of one month. Similar to the QOL fluctuations in all cases, both the mean FACT P-TOI scores and FACT P-Total scores by none, short term, and long term hormonal therapy had dropped to the bottom in the first or second month and is recovering quickly (Fig. 2). Until about the second year, the mean value was lowest in the long-term administration group than in the other two groups, and the mean value was about the same in the no- and the short-term administration group all the time both in FACT P-TOI and P-Total scores (Fig. 2).

**Fig. 3:**
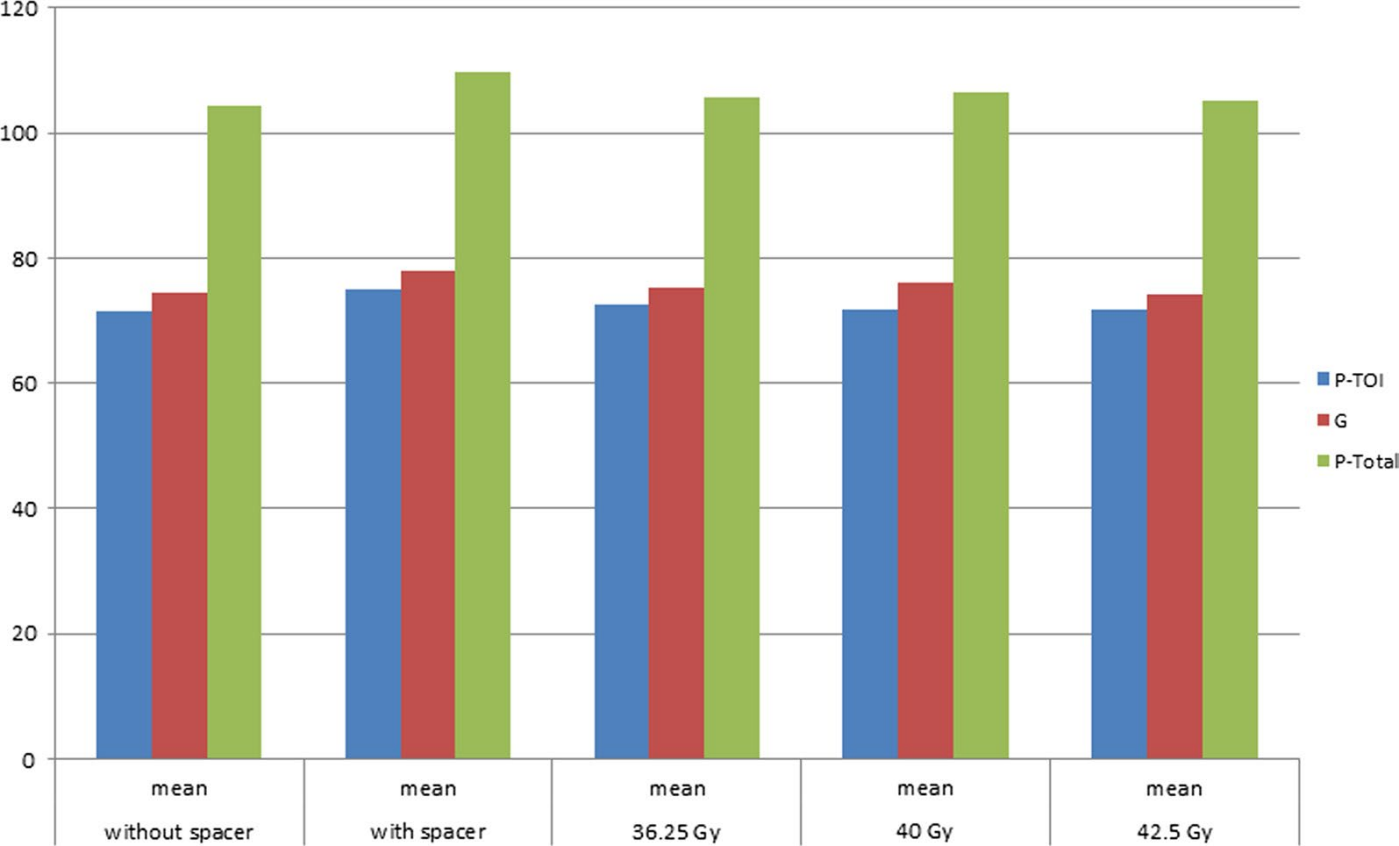
The mean score of FACT P-TOI, FACT-G, and FACT P-Total at 1 month divided with or without spacer and by total RT dose

With respect to use of a hydrogel spacer, the score of FACT P-TOI, FACT-G, and FACT P-Total at 1 month was 71.35 (SE: 1.02), 74.41 (0.96), and 104.26 (1.31) without spacer versus 74.97 (1.76) (*p* = 0.11 by unpaired t-test), 77.94 (1.69) (*p* = 0.10), and 109.74 (2.28) (*p* = 0.062) with spacer, respectively. With respect to the SBRT dose, the FACT P-TOI, FACT-G, and FACT-P-Total scores at 1 month were 72.58 (SE: 1.17), 75.22 (1.09), and 105.60 (1.46) after 36.25 Gy (*p* = 0.33 of 36.25 Gy versus 40 Gy, *p* = 0.68 of 36.25 Gy versus 42.5 Gy, and *p* = 0.99 of 40 Gy versus 42.5 Gy), and 71.77 (2.21), 76.05 (2.16), and 106.39 (3.02) (*p* = 0.71, 0.82, and 0.74) after 40 Gy, 71.85 (6.29), 74.15 (5.47), and 105.15 (7.88) (*p* = 0.79, 0.84, and 0.88) after 42.5 Gy, respectively (Fig. 3).

**Fig. 4:**
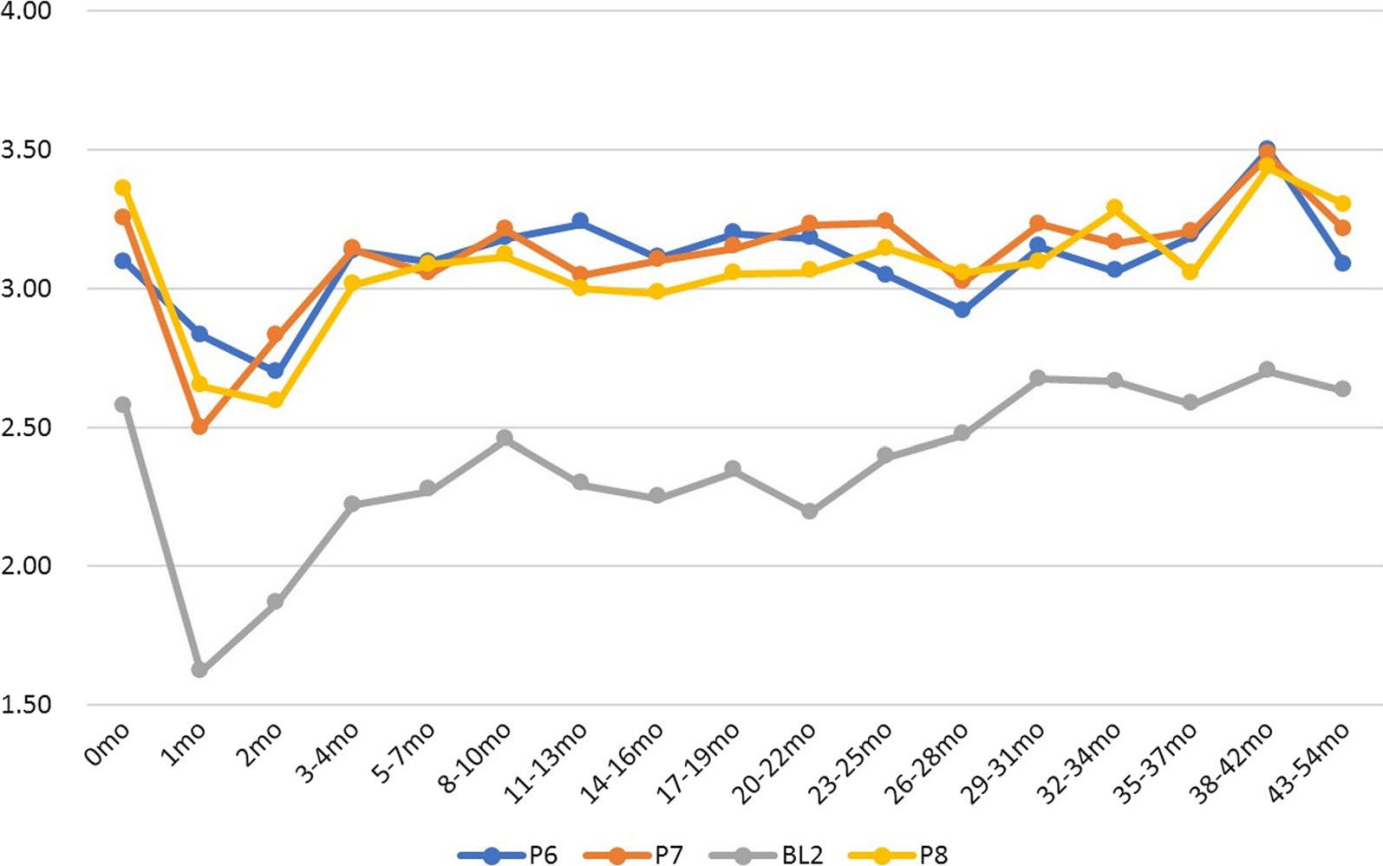
Mean four subscale scores. P6 (I have trouble moving my bowels): blue, P7 (I have difficulty urinating): orange, BL2 (I urinate more frequently than usual): gray, and P8 (My problems with urinating limit my activities): yellow line

P7 (I have difficulty urinating) (average score: 3.25 before SBRT, 2.49 at 1 month, and 3.14 at 2 months) and BL2 (I urinate more frequently than usual) (2.58, 1.62, and 2.22, respectively) were the lowest 1 month after the end of SBRT (both *p* < 0.0001) (Fig. 4). Improvement was observed at 2 months and was fully recovered by months 3–4. P6 (I have trouble moving my bowels) and P8 (My problems with urinating limit my activities) continued to decline until the second month and recovered by months 3–4 (Fig. 4).

**Fig. 5:**
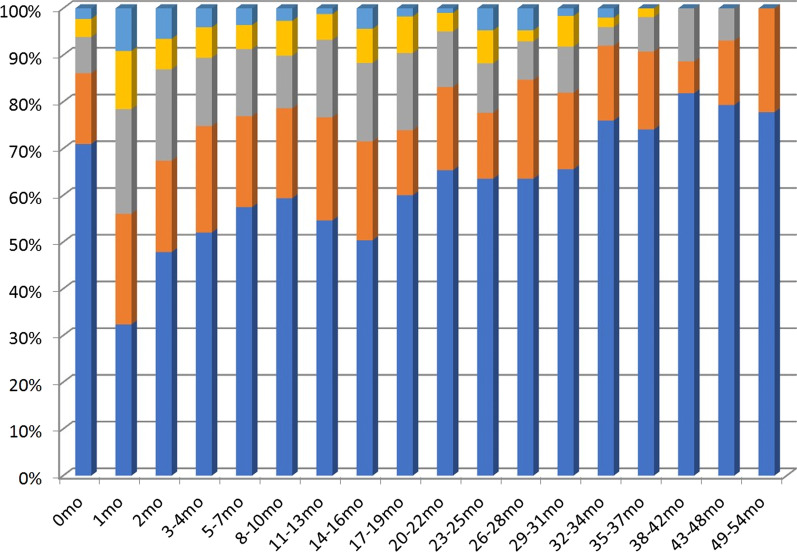
The distribution of answers to subscale GP5 (I am bothered by side effects of treatment (not at all: dark blue, a little bit: orange, somewhat: gray, quite a bit: yellow, and very much: sky blue)

The percentage of those who answered that they were suffering from the side effects of GP5 (I am bothered by side effects of treatment) was also the highest in the first month after SBRT at 22%. While this figure had increased from the 8% recorded during the pre-SBRT period, it then dropped to 11% by months 3–4 (Fig. 5). Subsequently, this percentage gradually decreased over time. By including those who were "somewhat,” bothered by side effects, this result changed from 15% before SBRT to 45% at the 1st month, 32% at the 2nd month, and 28% at 3–4 months.

## Discussion

In this retrospective study, we evaluated the QoL of 439 patients treated with SBRT for low-, intermediate-, and high-risk PC. To date, most QoL evaluations using FACT-P after SBRT for PC have only done so for metastatic cases [4]. However, since these studies did not irradiate the primary prostate lesion, their results cannot be compared with the QoL after radical SBRT. Using the FACT-P questionnaire, it is highly novel to see the time-series changes in QoL after radical PC SBRT. The FACT-P has also been used in many phase III studies on PC [5, 6, 13].

According to the previous reports [13–20], physician-reported acute GI and GU toxicity of grade 3–5 after SBRT of 2, 4, or 5 fractions were 0–2% and 0–2.5%, respectively. According to Kishan et al. [21], the crude incidence of acute grade 3–5 GU toxic events after SRT was 0.60% (13/2142 patients) and of GI toxic events was 0.09% (n = 2), and the 7-year cumulative incidence of late grade 3–5 GU toxic events was 2.4% (95% CI, 1.8%-3.2%) and of late grade 3–5 GI toxic events was 0.4% (95% CI,0.2%-0.8%). In our study, 45% of patients answered “somewhat” or more to GP5 (I am bothered by side effects of treatment) at 1 month after the last SBRT and 10–30% and 0–13% of patients answered “somewhat” or more and “quite a bit” or more in GP5 after 3–4 months, respectively. Moreover, 22% of patients responded that they were “quite a bit” or more troubled by the treatment’s side effects. In general, patient-reported side effects are more frequent than physician-reported side effects.

Previous studies of a systematic review have investigated the QoL change using FACT-P after conventional intensity-modulated radiation therapy (IMRT), but unlike our study, included an initial questionnaire 3 months after irradiation and found no decrease in QoL [22]. In contrast, we collected novel QoL data before SBRT, as well as during the first and second months after SBRT.

In a single-arm, phase II trial from multiple centers in Singapore [23], QoL of 74 men was assessed by EPIC at pretreatment, 12, and 24 months after SBRT of 36.25 Gy in 5 fractions. Overall, no significant change was observed in QoL scores over time across the three domains of urinary, bowel, and sexual scores [23]. The Dutch HYPO-RT-PC trial with the updated long-term QoL analyses at baseline, the end of radiotherapy, months 3, 6, 12, and 24 after radiotherapy, every other year thereafter up to 10 years, and at 15 years using the validated Prostate Cancer Symptom Scale (PCSS) and EORTC Quality-of-Life Questionnaire (QLQ-C30) confirmed that ultra-hypofractionated radiotherapy of 42.7 Gy in 7 fractions (n = 583) was equally well tolerated as conventionally fractionated RT of 78 Gy in 39 fractions (n = 582) up to 6 years after treatment [24]. According to Boyer et al. [22], the median AUASS (n = 60) increased from 4.5 points before SRT to 11 points while on SRT of 37 Gy in 5 fractions (*p* < 0.01), but was 5 points at 36 months after SRT (*p* = 0.65). These results were also consistent with the data after one year in the present study. According to Georgetown University’s report [11], which is the only existing study looking at QoL one month after SBRT of 35 Gy or 36.25 Gy in 5 fractions, the median baseline American Urological Association symptom score (AUASS) of 8 in 96 men significantly increased to 11 at 1 month (*p* = 0.001), but returned to baseline at 3 months (*p* = 0.60). Although a sole questionnaire after one month might have been really too short, it was conducted even in this survey by according to Georgetown University’s report [11]. According to Katz et al. [25], mean EPIC urinary (n = 262) and bowel QoL (n = 263) declined at 1 month post-SRT of 35–36.25 Gy in 5 fractions using Cyberknife technology, returned to baseline by 2 years, and remained stable thereafter and of patients potent at baseline evaluation, 67% remained potent at last follow-up with follow-up as long as 8 years. In a meta-analysis of curative-intent SBRT for localized PC [26], the EPIC-26 was used to assess 3,293 patients. Information for urinary, bowel, and sexual domain scales was readily extractable, and the data were pooled. The EPIC urinary and bowel scores returned to baseline by 2 years post-treatment (*p* = 0.90 and 0.09, respectively) and did not significantly differ at 5 years post-SBRT (*p* = 0.50 and 0.80, respectively), although these studies did not include early 1 or 2 month QoL measures. These results do not differ substantially from those of our study.

There are several points that could limit the strength of the study. First of all, there were times when questionnaire response rate was small, such as after the 38th month. Since we included all patients treated with SBRT during this period, only 71% of patients had a baseline assessment and 55% were evaluated at 1 month. Thus, our QoL assessments may follow a heterogeneous distribution. Secondly, we did not compare patient-reported outcome measures (PROMS) with physician reported outcomes nor toxicity scale of CTCAE in this study.

## Conclusions

QoL declined at 1 month after the end of prostate SBRT, then recovered, and returned to the same level as before treatment 3–4 months after SBRT. The decrease in QoL in the first month was particularly remarkable in patients who received long-term hormone injections. One month after the end of SBRT, about 22% of people were "quite a bit or very much" troubled by the side effects of the treatment. Our findings support that SBRT could be considered a standard radiotherapeutic strategy for localized PC.

## Data Availability

The datasets used and/or analyzed during the current study are available from the corresponding author on reasonable request.

## Abbreviations

AUASS: American Urological Association symptom score
CBCT: Cone-beam computed tomography
CT: Computed tomography
CTV: Clinical target volume
EORTC QLQ: European Organization for Research and Treatment of Cancer Quality-of-Life Questionnaire
EPIC: Expanded Prostate Cancer Index Composite
EWB: Emotional well-being
FACT-G: FACT-General
FACT-P: Functional Assessment of Cancer Therapy-Prostate
FDG: Fluorodeoxyglucose
FWB: Family well-being
GI: Gastrointestinal
GU: Genitourinary
HDR: High dose rate
HIFU: High-intensity focused ultrasound
HRQoL: Health related quality of life
IMRT: Intensity-modulated radiation therapy
LDR: Low dose rate
MRI: Magnetic resonance imaging
NCCN: National Comprehensive Cancer Network
PC: Prostate cancer
PCS: Prostate cancer subscale
PCSS: Prostate Cancer Symptom Scale
PET: Positron emission tomography
PROMS: Patient-reported outcome measures
PSA: Prostate-specific antigen
PSMA: Prostate specific membrane antigen
PTV: Planning target volume
PWB: Physical well-being
QoL: Quality of life
SBRT: Stereotactic body radiotherapy
SV: Seminal vesicle
SWB: Social well-being
TOI: Trial Outcome Index
VMAT: Volumetric modulated arc therapy
WHO: World Health Organization
